# Supplementary figures and images for: Manipulation of the unfolded protein response: A pharmacological strategy against coronavirus infection
Source: PLoS Pathog. 2021 Jun 17;17(6):e1009644. doi: 10.1371/journal.ppat.1009644 (PMC8211288; doi:10.1371/journal.ppat.1009644)

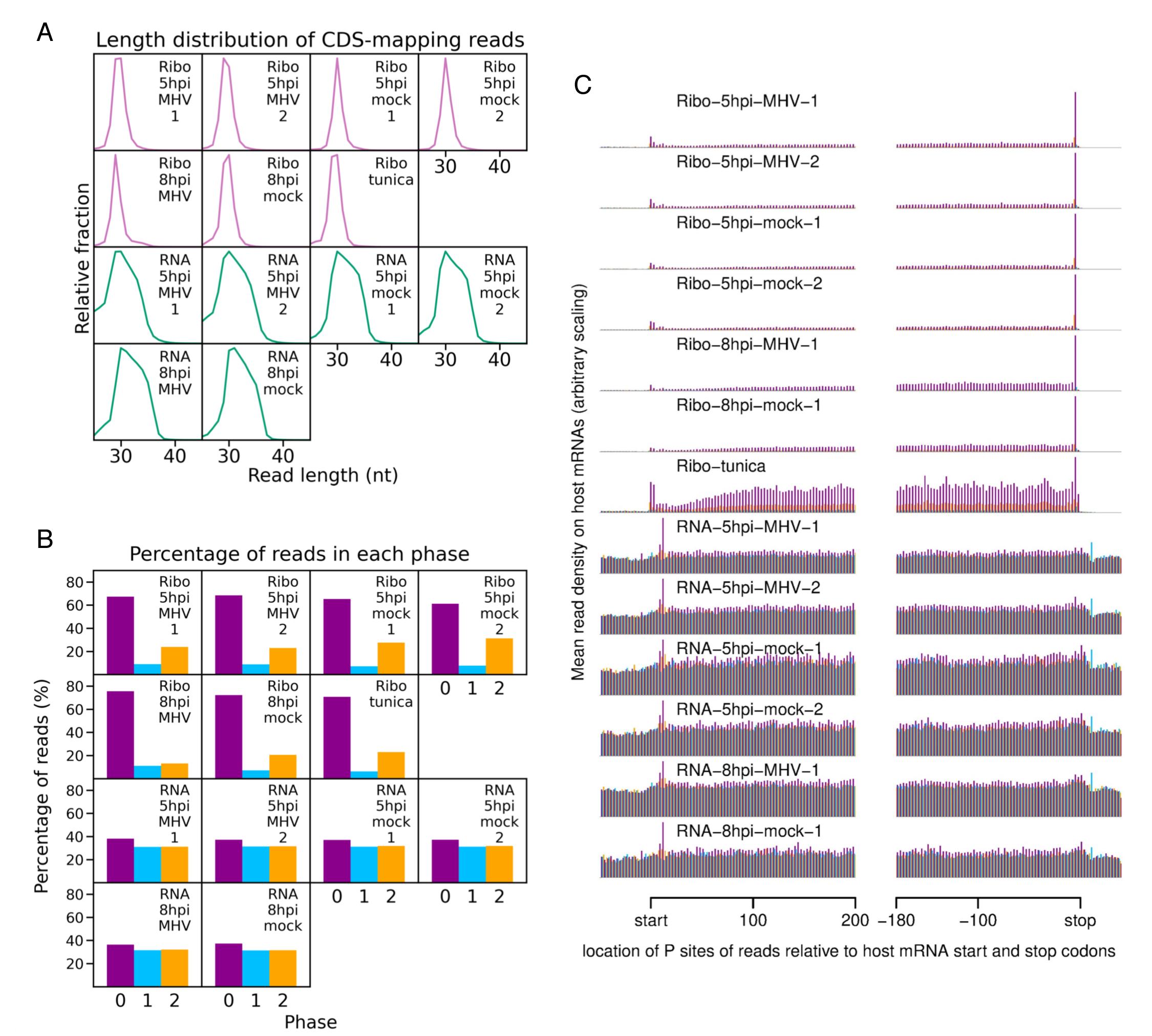

Supplement: S1 Fig — (A) Length distribution of positive-sense reads mapping within host CDSs. For RiboSeq libraries (pink) the characteristic sharp peak at 28–29 nt is observed, reflective of the length of mRNA protected from RNase I digestion. Read lengths of RNASeq libraries (green) are determined by alkaline hydrolysis and gel purification size selection (25–34 nt), leading to a broader length distribution. (B) Percentage of reads (all read lengths) attributed to each phase, for positive-sense reads mapping within host CDSs. Phases correspond to which position within the codon the 5′ end of the read maps to (0: purple, 1: blue, 2: yellow). The 5′ end coordinate of RiboSeq reads is influenced by the position of the translating ribosome, leading to a clear dominance of the 0 phase. For RNASeq reads the 5′ end coordinate is determined by alkaline hydrolysis so does not result in a dominant phase. (C) Distribution of host mRNA-mapping reads relative to start and stop codons. Only transcripts with an annotated CDS of at least 150 codons, 5′ UTR of at least 60 nt, and 3′ UTR of at least 60 nt were included in the analysis. The total number of positive-sense reads from all these transcripts mapping to each position was plotted, with an offset of +12 relative to the 5′ end coordinate to represent the inferred ribosomal P site. Although ribosomal P site position is not relevant to RNASeq reads, these were also plotted with a +12 nt offset to facilitate comparison. Data are coloured according to phase as in B. For RiboSeq libraries there is clear triplet periodicity visible across the CDS, reflective of the length of a codon, and a large peak corresponding to terminating ribosomes—characteristic of samples harvested without drug pre-treatment. Very few RiboSeq reads map to the UTRs (and particularly the 3′ UTR), indicating very little contamination of the mRNA fraction with non-ribosome-protected-fragment reads. As expected, for RNASeq libraries the coverage does not differ greatly between th [file ppat.1009644.s001.tif]

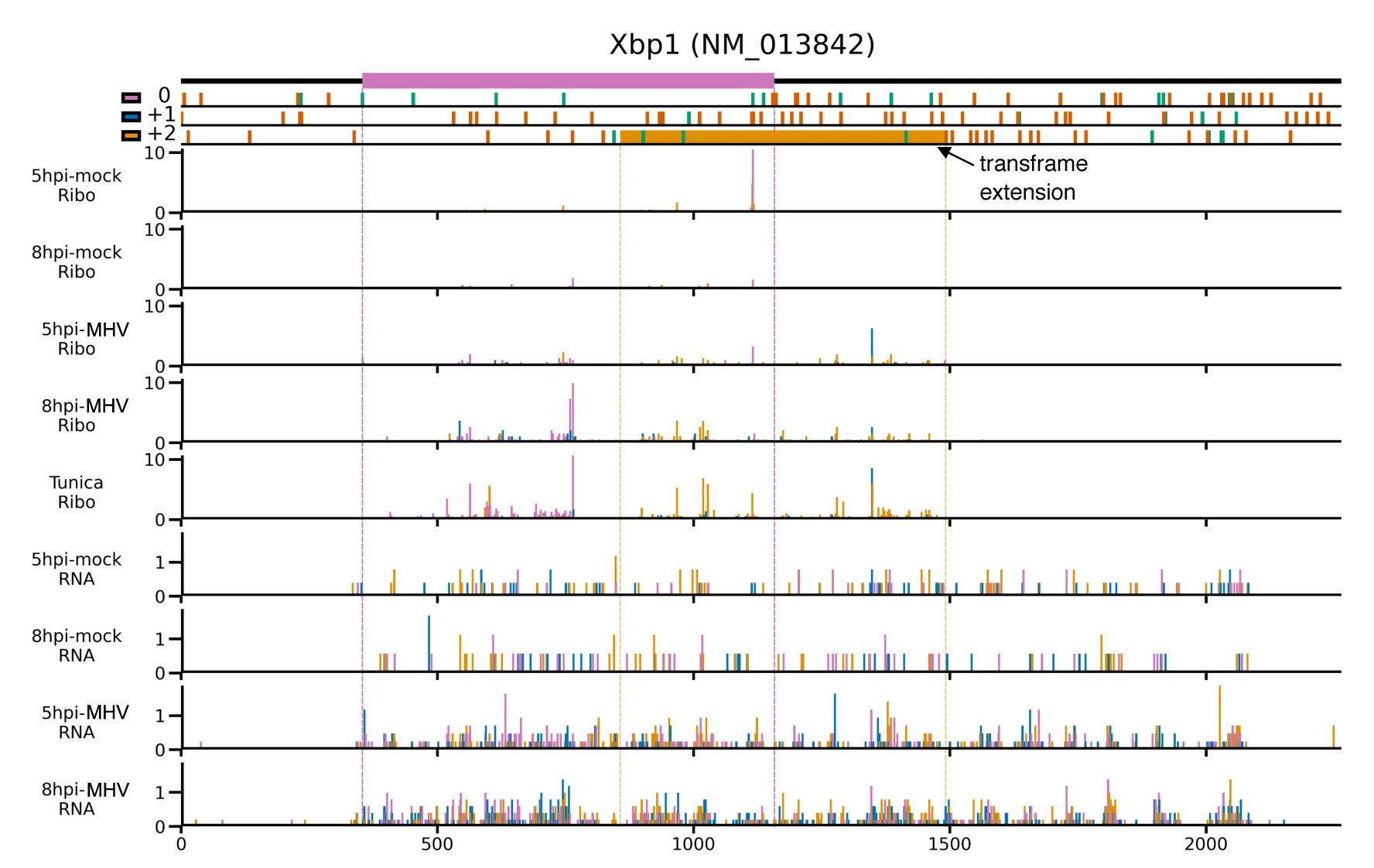

Supplement: S2 Fig — Analysis of RPFs (mock and MHV-infected samples plus tunicamycin-treated sample) and RNASeq reads (mock and MHV-infected samples) mapping to Xbp1-u (NCBI RefSeq mRNA NM_013842). Cells were infected with MHV-A59 or mock-infected and harvested at 5 h p.i. or 8 h p.i. (libraries from Fig 1D and 1E). One sample was treated with 2 μg/ml tunicamycin, a pharmacological inducer of all three branches of the UPR, as a positive control. Reads are plotted at the inferred position of the ribosomal P site and coloured according to phase: pink for 0, blue for +1, yellow for +2. The 5′ end position of RNASeq reads is not determined by ribosome position and therefore should not show a dominant phase. The main ORF (0 frame) is shown at the top in pink, with start and stop codons in all three frames marked by green and red bars (respectively) in the three panels below. The yellow rectangle in the +2 frame indicates the extended ORF that results from splicing by IRE1. Reads resulting mainly from translation of the spliced Xbp1-s isoform can be seen in yellow (+2 phase), downstream of the main ORF annotated stop codon. Dotted lines serve as markers for the start and end of the features in their matching colour. Read densities are plotted as reads per million host-mRNA-mapping reads. Bar widths were increased to 4 nt to aid visibility, and therefore overlap, and were plotted sequentially starting from the 5′ end of the transcript. (TIF) [file ppat.1009644.s002.tif]

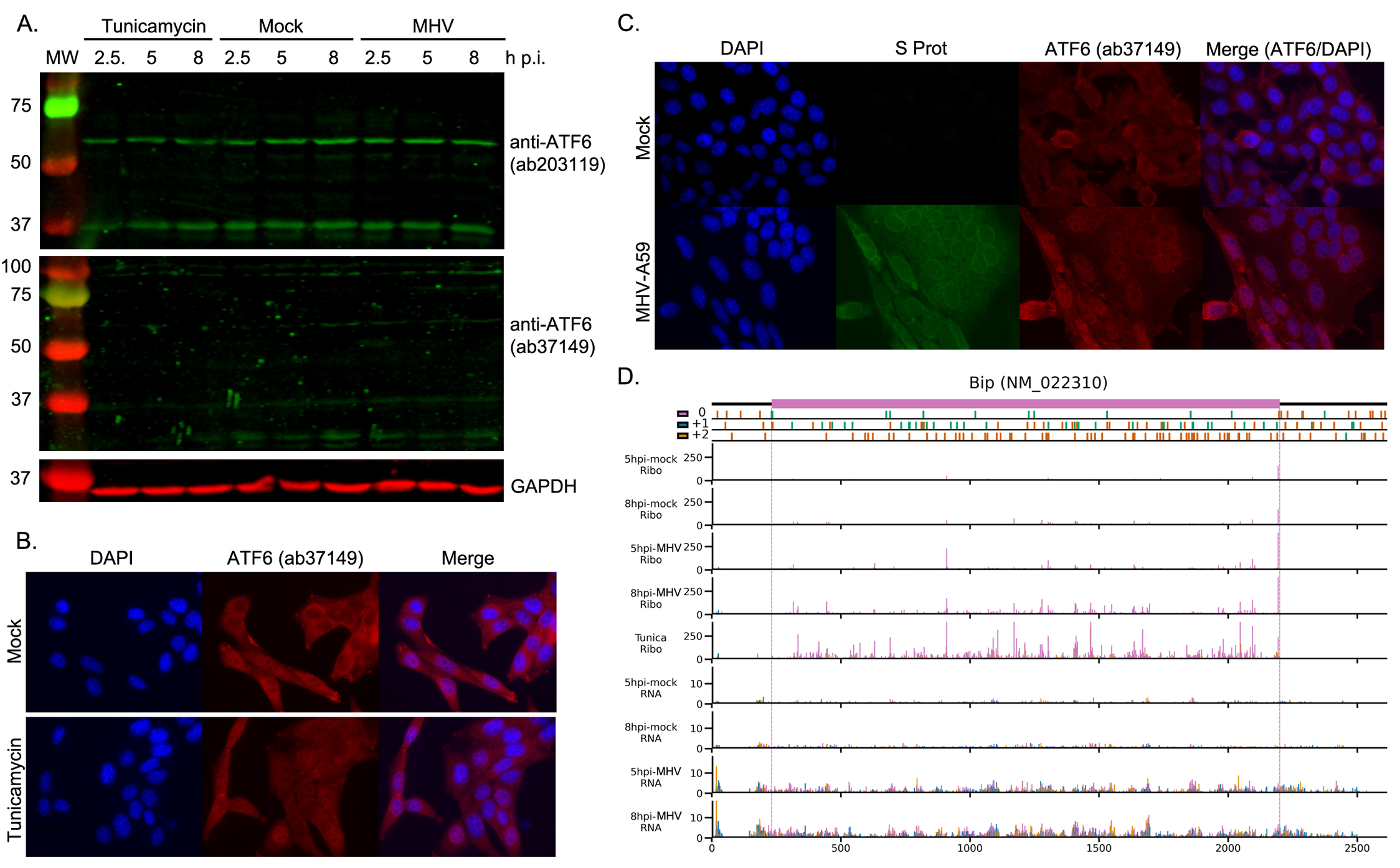

Supplement: S3 Fig — 17 Cl-1 cells were incubated in the presence of tunicamycin (2 μg/ml) or infected with MHV-A59 (MOI 5) and harvested at 2.5, 5 and 8 h. (A) Cell lysates were separated by 12% SDS-PAGE and immunoblotted using anti-ATF6 (1:1000, Abcam ab203119, upper), anti-ATF6 (1:1000, Abcam ab37149, middle). GAPDH was used as loading control. Representative images of fixed and permeabilised cells treated with tunicamycin for 6 h (B) or infected with MHV for 8 h (C) and incubated with anti-ATF6 (1:500, Abcam ab37149, red) and anti-S protein (green). Nuclei are counterstained with DAPI (blue). Images were taken in an Evos FLII microscope at 60X magnification. Scale bar: 100 μm. (D) Analysis of RPFs and RNASeq reads mapping to Bip (NM_022310). Plot constructed as described in S2 Fig. Note that in order to properly visualise RPFs across the ORF, the y-axis has been truncated at 400 reads per million host-mRNA-mapping reads for the RiboSeq samples, leaving some RPF counts for tunicamycin-treated cells and MHV-infected cells off-scale. (TIF) [file ppat.1009644.s003.tif]

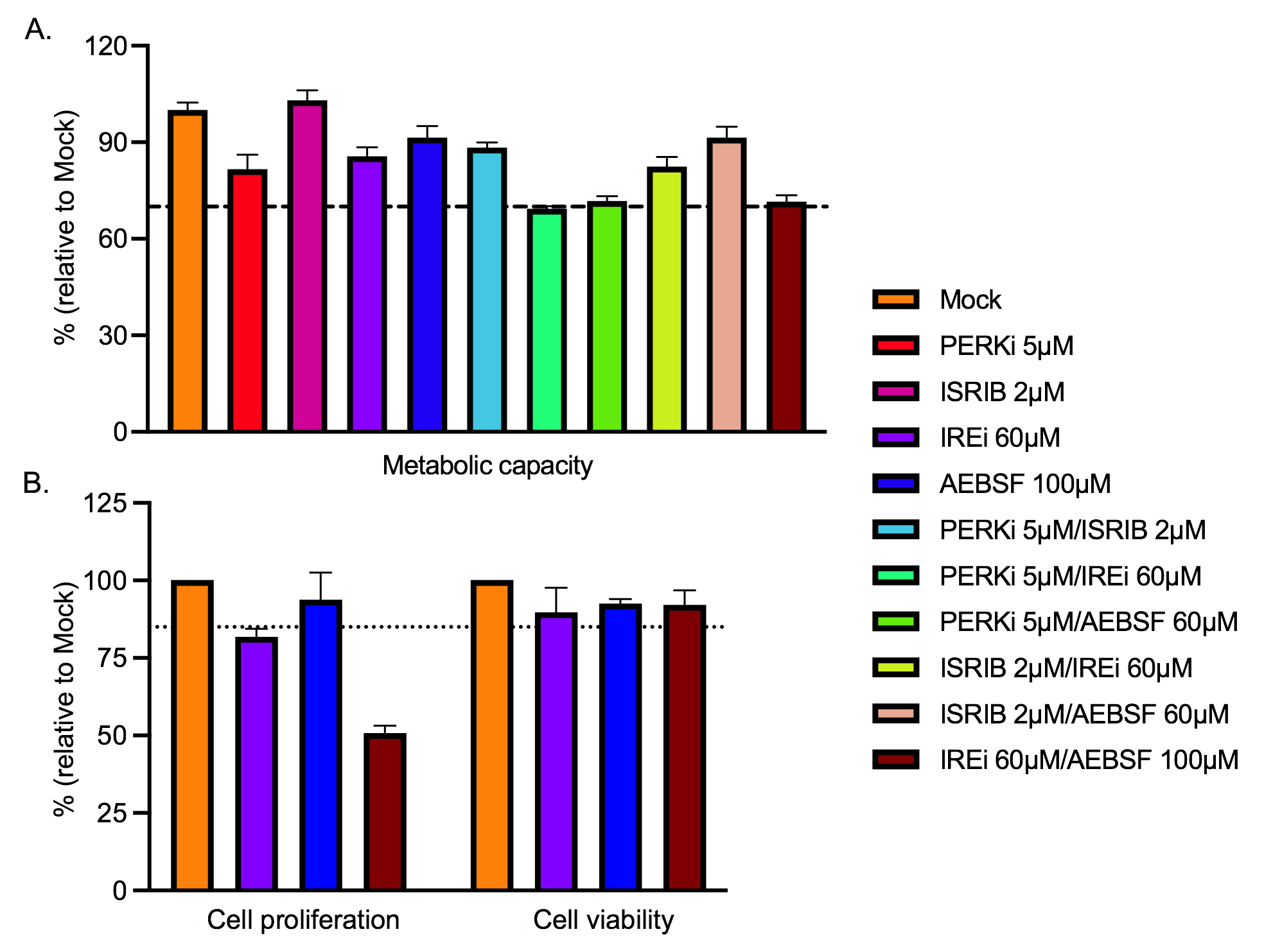

Supplement: S4 Fig — 17 Cl-1 cells were treated with the different UPR inhibitors at the indicated concentrations. Experiments were performed in triplicate using CellTiter-Blue Cell Viability Assay to assess metabolic capacity (dashed line represents 70% threshold) (A) and in duplicate using trypan blue exclusion assay to assess cell proliferation and viability (B) in treatment conditions involving IREi or AEBSF. Cell viability in all cases tested was greater than 85% (dotted line). Percentages are given relative to untreated cells. Error bars represent standard errors. (TIF) [file ppat.1009644.s004.tif]

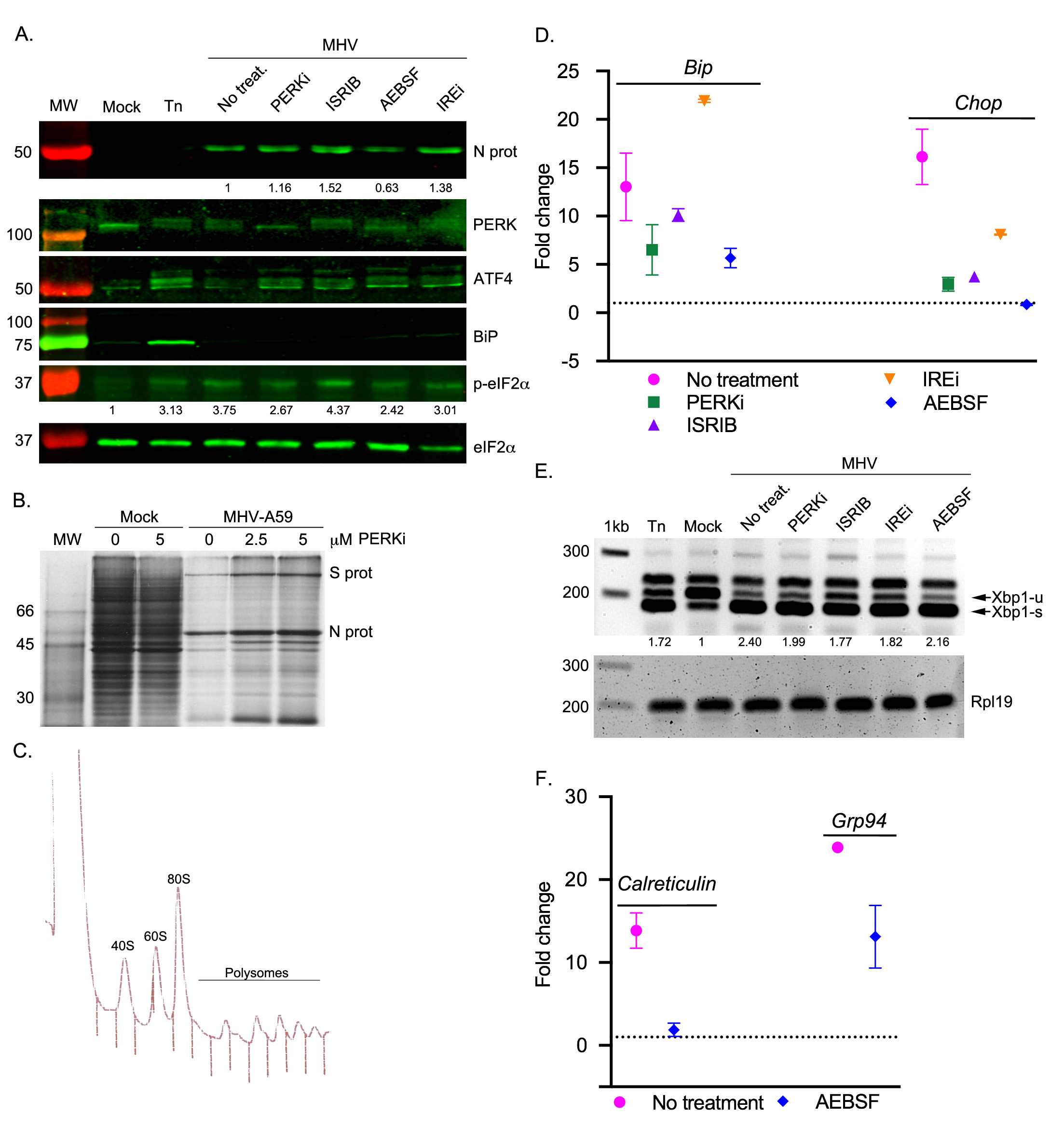

Supplement: S5 Fig — MHV-infected cells (MOI 5) were treated with UPR inhibitors (5 μM PERKi, 2 μM ISRIB, 60 μM IREi, or 100 μM AEBSF). The inhibitors were added to the cells immediately after the virus adsorption period and maintained in the medium until cells were harvested 8 h later. (A) Western blot analysis of MHV N protein, PERK, ATF4, BiP, p-eIF2α and eIF2α as loading control. Protein band quantifications (performed as described for Fig 2) are provided for MHV N protein and p-eIF2α and immunoblots are representative of three biological replicates. (B) 17 Cl-1 cells infected with MHV-A59 and treated with 0, 2.5 or 5 μM of PERKi were metabolically pulse-labelled with [35S]Met for 1 h at 5 h p.i. Cells were lysed just after pulse and subjected to 10% SDS-PAGE followed by autoradiography. (C) Polysome profiling as described in Fig 2C of MHV-infected cells at 5 h p.i. treated with 5 μM of PERKi. (D) RT-qPCR (performed as described in Fig 2B) of Bip and Chop mRNA from two biological replicates of MHV-infected cells treated with UPR inhibitors as described in Fig 2B. (E) RT-PCR analysis of Xbp1-u and Xbp1-s mRNAs, performed as described in Fig 2D. (F) RT-qPCR (performed as described in Fig 2B) of Calreticulin and Grp94 mRNA from two biological replicates of MHV-infected cells treated with UPR inhibitors as described in Fig 2B. (TIF) [file ppat.1009644.s005.tif]

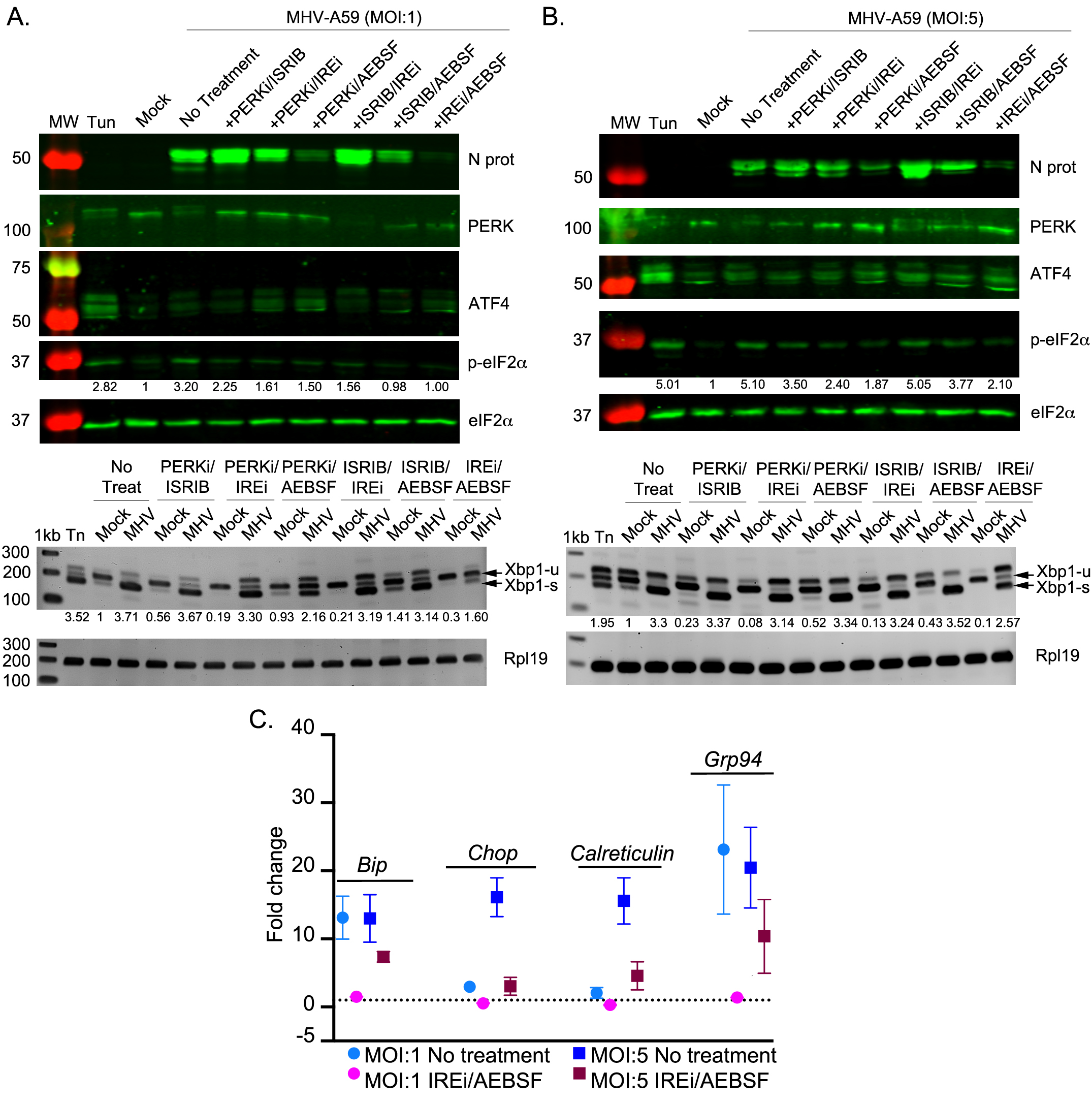

Supplement: S6 Fig — MHV-infected cells were treated with the combinations of the UPR inhibitors shown in Fig 3B. The inhibitors were added to the cells immediately after the virus adsorption period and maintained in the medium until cells were harvested 8 h later. Western blot analysis (upper) of MHV N, PERK, and p-eIF2α proteins from cell lysates of MHV-infected cells at MOI 1 (A) and at MOI 5 (B). The N protein panels have been duplicated from Fig 3C to facilitate comparison. Protein band quantifications, normalised by eIF2α as a loading control and given relative to the mock, are provided for p-eIF2α. RT-PCR analysis of Xbp1-u and Xbp1-s mRNAs (lower panels), performed as described in Fig 2D. Immunoblots and agarose gels are representative of three biological replicates. (C) RT-qPCR (performed as described in Fig 2B) of Bip, Chop, Calreticulin and Grp94 mRNA for three biological replicates of a timecourse of MHV infection under no-drug or IREi 60 μM/AEBSF 100 μM treatment conditions. (TIF) [file ppat.1009644.s006.tif]

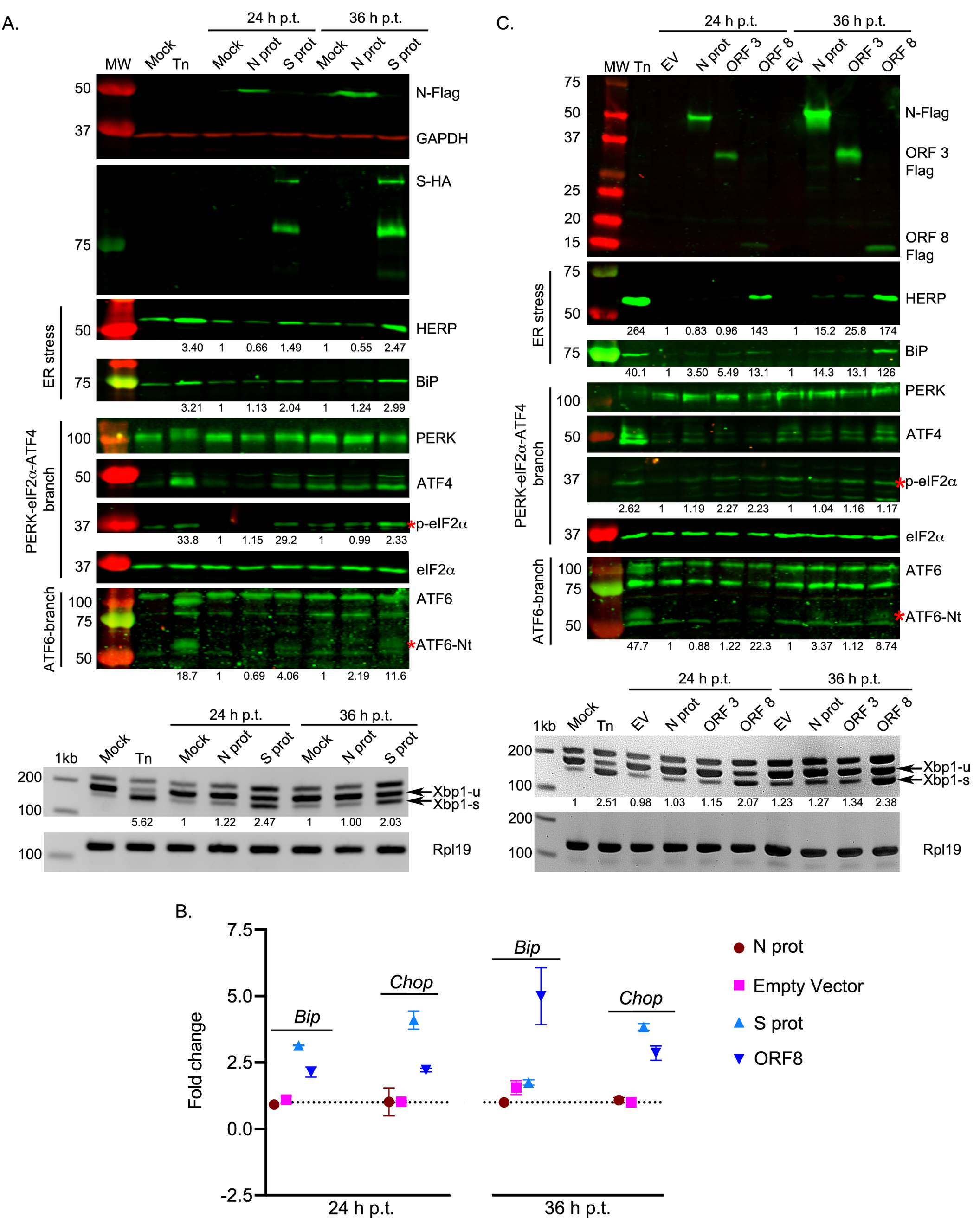

Supplement: S7 Fig — HEK-293T cells were treated with tunicamycin (Tn) or transfected with plasmids encoding SARS-CoV-2 S (S-HA), N (N-FLAG), ORF3 (ORF3-FLAG), ORF8 (ORF8-FLAG), pcDNA.3 as an empty vector (EV) control or mock-transfected as indicated. Cells were harvested at 24 and 36 h p.t. Western blot analysis (upper) of HERP, BiP, PERK, ATF4, p-eIF2α, eIF2α, ATF6, N-FLAG and either S-HA (A) or ORF3-FLAG and ORF8-FLAG (C). The specific p-eIF2α and ATF6-Nt bands are indicated with red asterisks. Protein band quantifications, normalised by eIF2α as a loading control and given relative to the mock, are provided for HERP, BiP, p-eIF2α and ATF6-Nt below the respective immunoblots. RT-PCR analysis of XBP1-u and XBP1-s mRNAs (lower), performed as described in Fig 2D. Immunoblots and agarose gels are representative of three biological replicates. (B) RT-qPCR of BIP and CHOP mRNA from two biological replicates of HEK-293T cells transfected with SARS-CoV-2 N, S or ORF8, or pcDNA.3 as an empty vector, and harvested at 24 and 36 h p.t. “h p.t.” = hours post-transfection. (TIF) [file ppat.1009644.s007.tif]

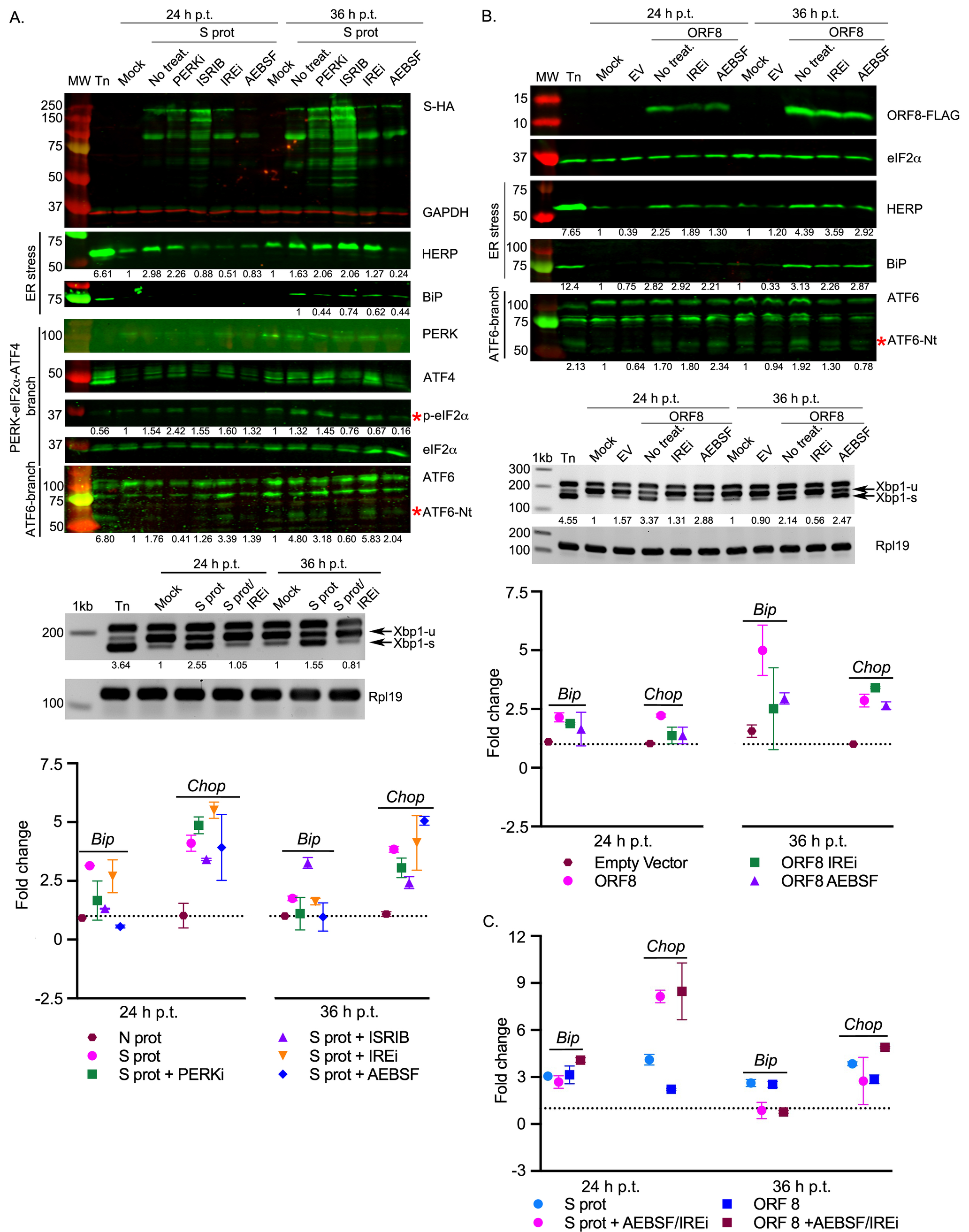

Supplement: S8 Fig — (A) HEK-293T cells were transfected with a plasmid encoding SARS-CoV-2 S (S-HA), mock-transfected, or treated with tunicamycin (Tn). At 8 h p.t., cells were treated with UPR inhibitors (5 μM PERKi, 2 μM ISRIB, 60 μM IREi or 100 μM AEBSF) and then harvested at 24 and 36 h p.t. Western blot analysis (upper) of S-HA, HERP, BiP, PERK, ATF4, p-eIF2α and ATF6. GAPDH and eIF2α are used as loading controls. The specific p-eIF2α and ATF6-Nt bands are indicated with red asterisks. Protein band quantifications, normalised to the loading controls and given relative to the mock, are provided for HERP, BiP, p-eIF2α and ATF6-Nt below the respective immunoblots. RT-PCR analysis using primers flanking the XBP1 splice site (middle panel), performed as described in Fig 2D. RT-qPCR (lower) of BIP and CHOP mRNA from two biological replicates of HEK-293T cells transfected and treated as described in panel A. Data are normalised as described in Fig 2B. (B) HEK-293T cells were transfected with a plasmid encoding SARS-CoV-2 ORF8 (ORF8-FLAG). At 8 h p.t., cells were treated with 60 μM IREi or 100 μM AEBSF and then harvested at 24 and 36 h p.t. Western blotting (upper), RT-PCR (middle) and RT-qPCR (lower) were performed as described in panel A. Immunoblots and agarose gels are representative of three biological replicates. RT-qPCR data of SARS-CoV-2 N, S, ORF8 and empty vector in panels A and B are reproduced from S7B Fig for comparison to the treated conditions, which were performed as part of the same experiment. (C) RT-qPCR of BIP and CHOP mRNA from two biological replicates of HEK-293T cells transfected with SARS-CoV-2 S and ORF8 harvested at 24 and 36 h p.t. Cells were treated with 60 μM IREi and 100 μM AEBSF or a no-drug treatment control. Data are normalised as described in Fig 2B. “h p.t.” = hours post-transfection. (TIF) [file ppat.1009644.s008.tif]

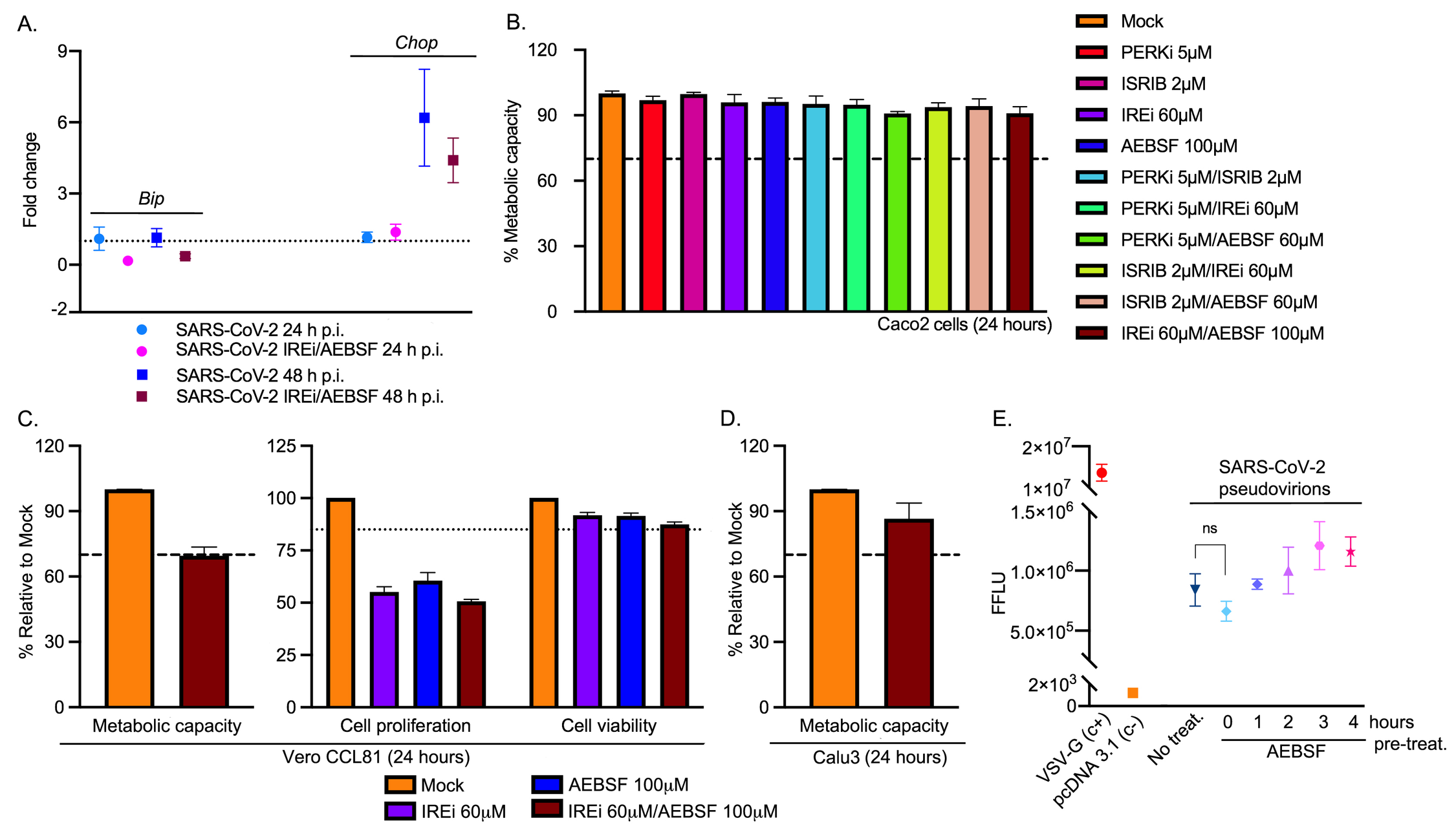

Supplement: S9 Fig — (A) RT-qPCR of BIP and CHOP mRNA from three biological replicates of Vero CCL81 cells infected and treated as described in Fig 5A. Data are normalised as described in Fig 2B. Caco2 (B), Vero CCL81 (C) or Calu3 cells (D) were treated with the different UPR inhibitors at the indicated concentrations for 24 h. Experiments were performed in triplicate using CellTiter-Blue Cell Viability Assay to assess metabolic capacity (B, C left panel and D) and in duplicate using trypan blue exclusion assay to assess cell proliferation and viability (C middle and right panels) in selected conditions. Percentages are given relative to untreated cells. (E) Infectivity of lentiviral particles pseudotyped with SARS-CoV-2 S protein. HEK-293T cells were transfected with ACE2 and TMPRSS2 and treated with 100 μM AEBSF between 0 and 4 h. Lentiviral particles engineered to contain a firefly luciferase reporter were pseudotyped with SARS-CoV-2 S, or vesicular stomatitis virus glycoprotein (VSV-G) as a positive control (c+, red) and empty pcDNA 3.1 vector as a negative control (c-, orange). Infectivity was measured as firefly luciferase units. Values show the mean averages of three biological replicates. Error bars represent standard errors. All t-tests are two-tailed and do not assume equal variance for the two populations being compared. “ns” = not significant. (TIF) [file ppat.1009644.s009.tif]

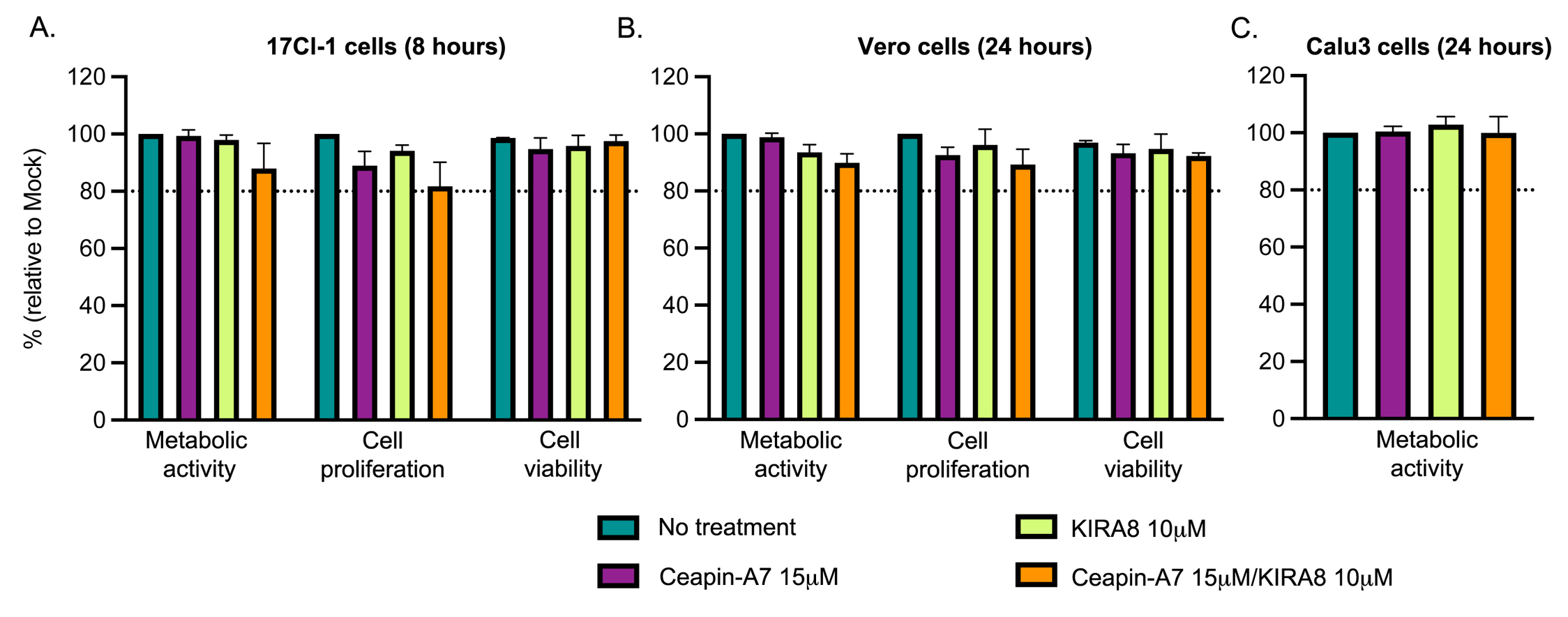

Supplement: S10 Fig — 17 Cl-1 (A), Vero CCL81 (B) or Calu3 cells (C) were treated with 10 μM KIRA8 or 15 μM Ceapin-A7 as individual treatments or in combination for 8 h (A) or 24 h (B-C). Experiments were performed in triplicate using CellTiter-Blue Cell Viability Assay to assess metabolic activity and in duplicate using trypan blue exclusion assay to assess cell proliferation and viability. Percentages are given relative to untreated cells and doted lines represent 80% of the mock value. (TIF) [file ppat.1009644.s010.tif]

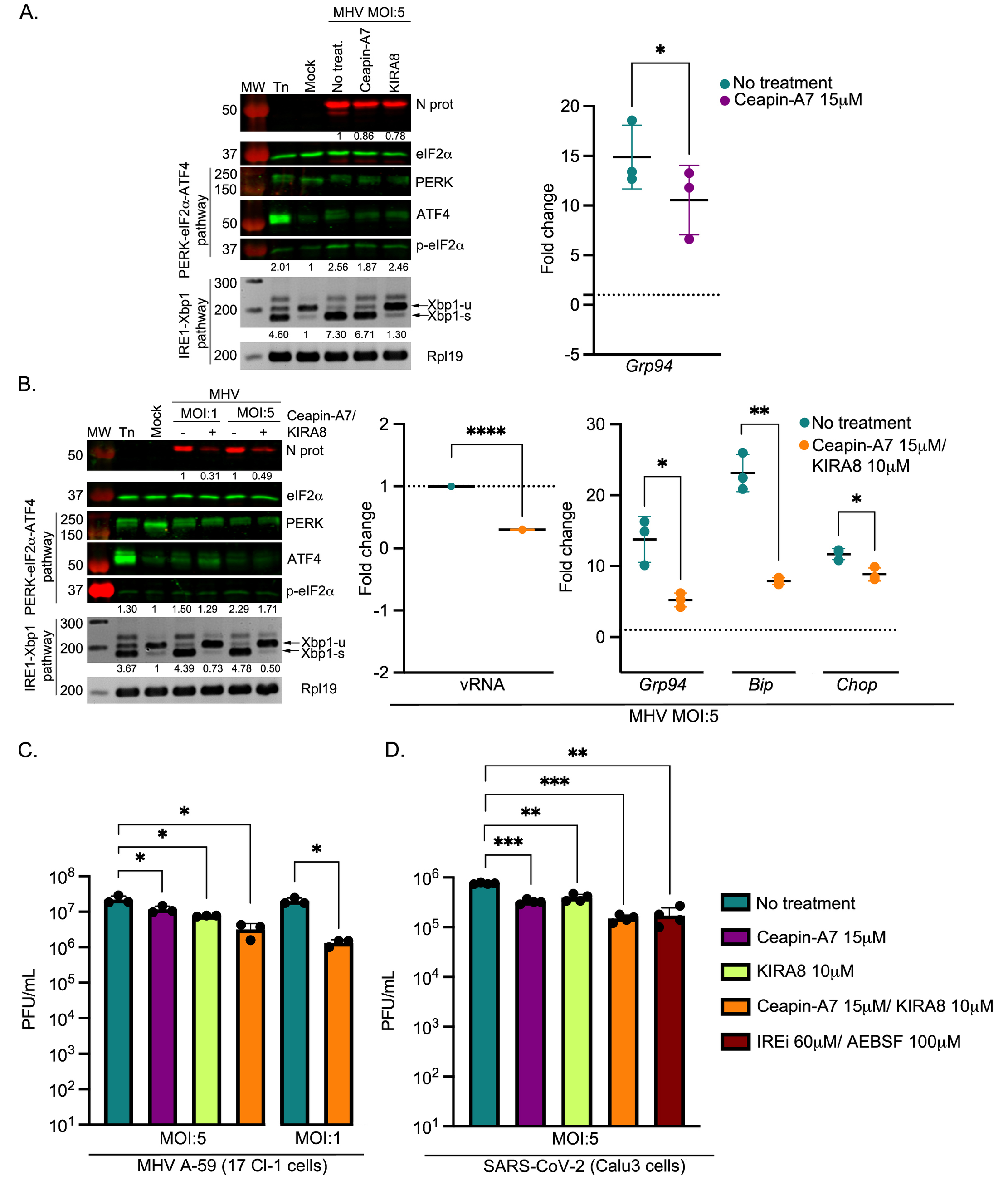

Supplement: S11 Fig — Western blot analysis (A and B upper left panels) of MHV N, PERK, ATF4 and p-eIF2α from cell lysates of MHV-infected cells (MOI 5) treated with 10 μM KIRA8 or 15 μM Ceapin-A7 as individual treatments (A) or in combination (B). The inhibitors were added to the cells immediately after the virus adsorption period and maintained in the medium until cells were harvested 8 h later. Protein band quantifications, normalised by eIF2α as a loading control and given relative to the mock, are provided for MHV N and p-eIF2α below the respective immunoblots. RT-PCR analysis of Xbp1-u and Xbp1-s mRNAs (A and B lower left panels) was performed as described in Fig 2D. RT-qPCR of Grp94 mRNA (A) or MHV vRNA, Grp94, BiP and Chop (B), performed for three biological replicates and normalised as described in Fig 2B. Immunoblots and agarose gels are representative of three biological replicates. (C) Plaque assays were performed with serial dilutions of the supernatant containing released virions from (A) and (B). (D) Viral titres of Calu3 cells infected with SARS-CoV-2 at MOI 5 and treated with the different UPR inhibitors a described in Fig 6. Plaque assays were performed with serial dilutions of the supernatant containing released virions harvested at 24 h p.i. In all cases, values show the mean averages of the titration of three biological replicates. Error bars represent standard errors. All p-values are from comparisons with the respective untreated control, with *p < 0.05, ** p < 0.01, *** p < 0.001 and **** p < 0.0001. (TIF) [file ppat.1009644.s011.tif]
