# Supplementary material for: Manipulation of the unfolded protein response: A pharmacological strategy against coronavirus infection
Source: PLoS Pathog. 2021 Jun 17;17(6):e1009644. doi: 10.1371/journal.ppat.1009644 (PMC8211288; doi:10.1371/journal.ppat.1009644)
Supplement: S5 Table — (DOCX) [file ppat.1009644.s016.docx]

**Supplementary Table 5. List of oligonucleotides used.** Note *fwd* indicates forward primer, and *rev* indicates reverse primer.

| **Name** | **Sequence (5′-3′)** |
| --- | --- |
| *Rpl19* *fwd* (mouse) | ATGCCAACTCCCGTCAGCAG |
| *Rpl19* *rev* (mouse) | TCATCCTTCTCATCCAGGTCACC |
| *Bip* *fwd* (mouse) | CCTGCGTCGGTGTGTTCAAG |
| *Bip* *rev* (mouse) | AAGGGTCATTCCAAGTGCG |
| *Chop* *fwd* (mouse) | ACGGAAACAGAGTGGTCAGTGC |
| *Chop* *rev* (mouse) | CAGGAGGTGATGCCCACTGTTC |
| *Xbp1* *fwd* (mouse) | GAACCAGGAGTTAAGAACACG |
| *Xbp1* *rev* (mouse) | AGGCAACAGTGTCAGAGTCC |
| *Gadd34* *fwd* (mouse) | GACCCCTCCAACTCTCCTTC |
| *Gadd34* *rev* (mouse) | TCTCAGGTCCTCCTTCCTCA |
| *Calreticulin* *fwd (mouse)* | TGTTACCAAGGCTGCAGAGA |
| *Calreticulin* *rev (mouse)* | GGCCTCTACAGCTCATCCTT |
| *Grp94* *fwd (mouse)* | AGTCGGGAAGCAACAGAGAA |
| *Grp94* *rev (mouse)* | TCTCCATGTTGCCAGACCAT |
| *Human RPL19 fwd* | ATGTATCACAGCCTGTACCTG |
| *Human RPL19 rev* | TTCTTGGTCTCTTCCTCCTTG |
| *Human BIP fwd* | CGGGCAAAGATGTCAGGAAAG |
| *Human BIP rev* | TTCTGGACGGGCTTCATAGTAGAC |
| *Human CHOP fwd* | ACCAAGGGAGAACCAGGAAACG |
| *Human CHOP rev* | TCACCATTCGGTCAATCAGAGC |
| *Human XBP1 fwd* | TTACGAGAGAAAACTCATGGC |
| *Human XBP1 rev* | GGGTCCAAGTTGTCCAGAATGC |
| *MHV N transcript fwd* | GCAATTACTGCCCAGATGGT |
| *MHV N transcript rev* | CTAGCAGGTGCAGACCTTCC |
| *SARS-CoV-2 N transcript fwd* | TCACCGCTCTCACTCAACAT |
| *SARS-CoV-2 N transcript rev* | CTGGCCCAGTTCCTAGGTAG |
