# Supplementary material for: Manipulation of the unfolded protein response: A pharmacological strategy against coronavirus infection
Source: PLoS Pathog. 2021 Jun 17;17(6):e1009644. doi: 10.1371/journal.ppat.1009644 (PMC8211288; doi:10.1371/journal.ppat.1009644)
Supplement: S1 Text — (PDF) [file ppat.1009644.s018.pdf]

Supplementary Materials and Methods:

Immunofluorescence

Cells seeded on top of glass coverslips were fixed using ultra-pure, methanol-free formaldehyde (Polysciences) at a final concentration of 4% and permeabilised with Triton (0.3% diluted in PBS). Subsequently, cells were blocked with 5% FCS for 10 min prior to primary antibody incubation. Coverslips were probed with primary antibodies diluted in 1% FCS at the recommended concentrations for 1 h at room temperature, followed by incubation with secondary antibodies for 1 h in the dark. Secondary antibodies were donkey anti-rabbit or goat anti-mouse antibodies conjugated to Alexa Fluor 488 or Alexa Fluor 594 (ThermoFisher Scientific). Nuclei were counterstained with DAPI (1 µg/mL, Sigma-Aldrich) for 5 min. Coverslips were mounted onto microscope slides with Prolong Gold Antifade mountant (ThermoFisher Scientific) and allowed to cure overnight at room temperature. Cells were then imaged using an Evos FL Auto 2 microscope (ThermoFisher Scientific) with 60X oil-immersion objective. All images were exported as TIFF files.

Metabolic labelling: 17 Cl-1 cell monolayers were infected with MHV-A59 at a MOI of 5 PFU/cell. At 5 h p.i., cells were washed twice with PBS and labelled for 1 h in methionine-free DMEM supplemented with 125 µCi/ml [^{35}S] methionine. After this period, cells were harvested, washed twice with PBS and resuspended in lysis buffer (50 mM Tris pH 7.5, 100 mM NaCl, 5 mM EDTA, 0.5% NP40). Cell lysate aliquots were mixed with Laemmli's sample buffer to a final concentration of 1X and subjected to 10% SDS-PAGE followed by autoradiography.
