# Supplementary material for: Manipulation of the unfolded protein response: A pharmacological strategy against coronavirus infection
Source: PLoS Pathog. 2021 Jun 17;17(6):e1009644. doi: 10.1371/journal.ppat.1009644 (PMC8211288; doi:10.1371/journal.ppat.1009644)
Supplement: Attachment — Submitted filename: renamed_65999.pdf [file ppat.1009644.s019.pdf]

Echavarría-Consuegra et al.: Responses to Reviewers' Questions

Dear Editor,

We are very grateful to the reviewers for their expert engagement with the work, their constructive criticisms and their suggestions on how to improve the manuscript. We have responded below to the questions point-by-point and also included several additional experiments with more specific inhibitors of the UPR (i.e. Ceapin-A7 and KIRA8, described in the new section of the manuscript starting at line 434) that provide further support for our findings, conclusions and mechanistic interpretations.

Yours faithfully,

Dr Nerea Irigoyen

on behalf of the authors

Part I – Summary

Reviewer #1: In this section, the reviewer had only one comment:

The paper provides relevant information regarding host–virus interactions at the transcriptional and translational level. However, there are some methodological concerns that should be addressed.

This comment is addressed in our responses to specific comments in Part II and Part III.

Reviewer #2: This reviewer interspersed a number of comments in this section. These are dealt with here, or where appropriate, in the responses to specific comments in Parts II and III.

(1) Overall, the effects on viral titers are very modest with the highest reduction (IRE1i/AEBSF) less than 100-fold (Fig. 3B) and the single inhibitors at most 10-fold.

Although the reductions in viral titre upon treatment with single inhibitors are relatively low in the case of MHV, they have a synergistic effect when added in combination reaching almost ~100-fold reduction (i.e. 99% decrease in virus release). In all these cases, these numbers are statistically significant, which provided the motivation to further explore these results and test these inhibitors on SARS-CoV-2. Greater reductions in titre were observed upon treatment of SARS-CoV-2-infected cells, and these experiments provide evidence that the UPR represents a potential antiviral target to combat this important human pathogen.

(2) *One concern is that several of the inhibitors exert cytopathic, or at least cytostatic effects (Fig. S4). In particular, the IRE1i/AEBSF combination reduces cell proliferation by ~40%, so one is left to wonder whether some of the reduction in MHV titers could simply be attributed to slower cell proliferation or cytotoxic effects. Aside from a short sentence pointing to Fig. S4, the authors do not discuss this data further, which would be important to address.*

This is now been addressed in Part II as a response to comment 5 for Reviewer 1 and comment 5 for Reviewer 2.

(3) *AEBSF is a highly promiscuous arylsulfonyl chloride serine protease inhibitor. It prevents ATF6 activation by inhibiting site-1-protease (S1P). Besides ATF6 cleavage, S1P has other important functions, including cleavage of sterol regulatory element-binding protein (SREBP). Therefore, inhibition of AEBSF could have a variety of pleiotropic effects and the antiviral activity could be entirely unrelated to ATF6 inhibition. In addition, inhibition of other serine (or cysteine) proteases by AEBSF could be relevant during MHV or SARS-CoV-2 infections, including the potential inhibition of viral 3Cpro or PLpro proteases. The only other protease excluded as a potential off-target of AEBSF is TMPRSS2, which is required for SARS-CoV-2 entry. Considering that the authors tie the reduction in viral replication to UPR inhibition, it would especially be important to validate that the effect is directly linked to ATF6 inhibition. One easy way to address this would be to use a more selective pharmacologic ATF6 inhibitor Ceapin-A7 (see Gallagher et al., Elife, 2017 (5), e11878), which acts upstream of S1P cleavage.*

New experiments have been performed to address this concern. This includes a new section in the manuscript starting at line 434 and Fig 6, S10 and S11 Figures.

Part II – Major Issues: Key Experiments Required for Acceptance

Reviewer #1:

(1). *RNAseq and Riboseq analysis of MHV infected cells was performed at 5 h post-infection. This time point seems to be too early to see the effect of expression of viral proteins on the cell. In fact, Fig. 2B shows at 5 hpi very low levels of Chop and Gadd34 mRNA, suggesting that the UPR was not activated yet, and other experiments in the paper were performed at 8 hpi.*

The RNASeq and RiboSeq experiments were performed as an initial, unbiased broad screen to identify pathways that were a prominent part of the host response to coronavirus infection. Translation of the MHV genome is already evident by ribosome profiling by 1 h p.i. (Irigoyen et al., 2016, PMID: 26919232, Fig 7). By 5 h p.i., massive amounts of viral replication, sgRNA production and viral ORF translation have occurred, and levels do not increase much further at 8 h p.i. (Irigoyen et al., 2016, PMID: 26919232, Fig 3), with a cytopathic effect becoming extensive by 10 h p.i. Therefore, 5 h p.i., at which time viral proteins are readily detectable by western blot (e.g. Fig 2A), was selected for the differential gene expression to identify host responses triggered by the mid-late stage of the viral replication cycle.

RiboSeq/RNASeq is expected to be more sensitive than western blot and qPCR, which may explain the more robust detection of UPR activation at this earlier timepoint, however, we also observed UPR activation at 5 h p.i. with other assays (e.g. eFI2α and PERK phosphorylation in Fig 2A by western blotting, *Xbp1* splicing in Fig 2D by RT-PCR, and an increase in transcription of *Chop* [~5.5-fold], *Gadd34* [~3.4-fold], *Bip* [~10-fold], *Calr* [~8-fold] and *Grp94* [~9.5-fold] in Fig 2B and Fig 2E by qPCR). We considered that western blotting, RT-PCR and qPCR were more appropriate, and less expensive, to further explore the time course of UPR activation. Collectively, these assays revealed that the UPR was further increased at 8 h p.i., hence the selection of this later timepoint for some subsequent experiments (e.g. Fig 3).

2. *Only SARS-CoV-2 S, N, 3a and 8 proteins were assayed to study the activation of the UPR. The rationale to exclude other viral proteins, such as E, M or accessory 6 and 7 proteins should be provided.*

We have expanded the paragraph beginning at line 315 to include a discussion of our rationale for this.

3. Fig S7. The activation of IRE1 pathway by S protein is clearly shown by the increase in Xbp-s RNA. However, the activation of ATF6 by S protein at 36 hpt is not that evident, since the band of ATF6-Nt is not too different from that of Mock and N. In fact, in S7B, the level of Bip expression in S-transfected cells at 36 hpt is similar to the empty vector.

We apologise for any confusion that this figure panel may have caused. Based on the original panel, it is possible that the reviewer mistook ATF6-Nt for a background band located just below. We have now made the location of the ATF6-Nt band clearer by indicating its position with a red asterisk. It is clear in the newly labelled panel that ATF6-Nt is highly activated. Indeed, we have now quantified by densitometry the ATF6-Nt protein band in Fig S7A. The relative amount of ATF6-Nt reflects the ratio of this protein band relative to the loading control (i.e. eIF2α), normalised against the mock lane at that specific time point. In this case, the fold-change of ATF6-Nt in S-transfected cells at 36 h p.t is 11.6 relative to the mock, which is considerably greater than the fold change of 2.19 in the N-transfected cells at 36 h p.t.

As the reviewer correctly indicates, *Bip* mRNA expression in S-transfected cells at 36 h p.t. is similar to N and the empty vector, whereas quantification of BiP in the western blot is ~3-fold greater than in the mock lane. As mentioned in the manuscript in line 232 (Fig 1A, Lee et al., 2003, PMID 14559994; and Luo et al., 2003, PMID 12871976), *Bip* transcription can be regulated by the PERK and IRE1α branches of the UPR as well as ATF6. The reduction in *Bip* transcript abundance seen at 36 h p.t. (compared to 24 h p.t.) may be the result of attenuation of the PERK branch, which results from GADD34 activation (note that the ratio of p-eIF2α at 36 h p.t. is lower than at 24 h p.t.).

4. Fig. 5B. Caco2 cells were used to analyze the relevance of the UPR in SARS-CoV-2 infection. The lung cell line Calu3, also used in Fig. 5C, would be a more physiological experimental model, since SARS-CoV-2 is mainly a respiratory virus.

As indicated by the reviewer, Caco2 and Calu3 cells, biologically relevant cell types for SARS-CoV-2 infection, were used alongside Vero CCL81 cells to assess the impact of different UPR inhibitors on SARS-CoV-2 replication. In a new Figure (5B) we describe UPR activation by SARS-CoV-2 (MOI:1) at 24 h p.i. in Calu3 cells. Confirming our findings from Vero CCL81 cells, the three UPR pathways were also activated by SARS-CoV-2 infection of Calu3 cells. A description of these results has also been added to the text, lines 383-386.

5. Fig. 5. All the experiments evaluating the antiviral effect of UPR inhibitors should be accompanied by cytotoxicity assays to exclude the impact of cell viability on viral production.

The cytotoxicity of the different compounds in Vero CCL81, Caco2 and Calu3 cells have now been fully assessed in CellTiter-Blue (to quantify the cellular metabolic activity) and trypan blue (to assess cellular proliferation and viability) assays. Some of these data are presented as before in S4 Figure and in addition, the cytotoxicity assays of Vero CCL81, Caco2 and Calu3 cells in the presence of inhibitors (described in Figure 5) have been moved to the S9 Figure. Furthermore, data related to the new UPR inhibitors that we have tested (i.e. Ceapin-A7 and KIRA8; see below) as described in Figure 6 have been added to S10 Figure.

In all experiments assessing the effect of UPR inhibitors on virus production, the drugs were only used at concentrations where metabolic activity and cell viability of the treated cells were at least 70% and 85%, respectively. This is consistent with many other drug studies on SARS-CoV-2 infection (Xiong et al., 2021, PMID: 33613281; and Salgado-Benvindo et al., 2020, PMID: 32513797). In particular examples where metabolic activity approached this threshold

(IREi/AEBSF in Vero cells or 17 Cl-1 cells), experimental outcomes were verified using the new inhibitors KIRA8 (IRE1α inhibitor) and Ceapin-A7 (ATF6 inhibitor). The latter inhibitors were well tolerated in cytotoxicity assays (metabolic activity at least 90% compared to untreated cells as indicated in S10 Figure), and also caused significant reductions in virus titre of up to 15-fold for MHV in 17 Cl-1 cells and up to ~500-fold for SARS-CoV-2 in Vero cells (Fig 6 and S11). The data presented in these newly added figures in the manuscript confirms that the inhibitory effect on virus production is due to UPR inhibition as opposed to cytotoxicity.

6. Lines 394-395. A significant reduction in the expression of MHV N protein (Fig 3C) and SARS-CoV-2 S protein (Fig 5A) is observed in the presence of UPR inhibitors. As discussed, this observation might have different interpretations that should be addressed. First, as indicated above, cytotoxicity caused by the inhibitors should be excluded. Then, an indirect inhibitory effect on viral early replication should be analyzed by measuring the viral RNA synthesis at early times post-infection.

As detailed in the previous comment, cytotoxicity of the UPR inhibitors has been assayed (S4, S9 and S10 Figures) and is not responsible for the observed effect. The newly included inhibitors Ceapin-A7 and KIRA8 are well tolerated in Vero cells (at least 90% metabolic activity, cell proliferation and cell viability compared to untreated cells; S10B Fig), and treatment of SARS-CoV-2-infected Vero cells with the Ceapin-A7/KIRA8 combination resulted in similar reductions in S protein levels to those seen with the original IREi/AEBSF combination (Fig 6A).

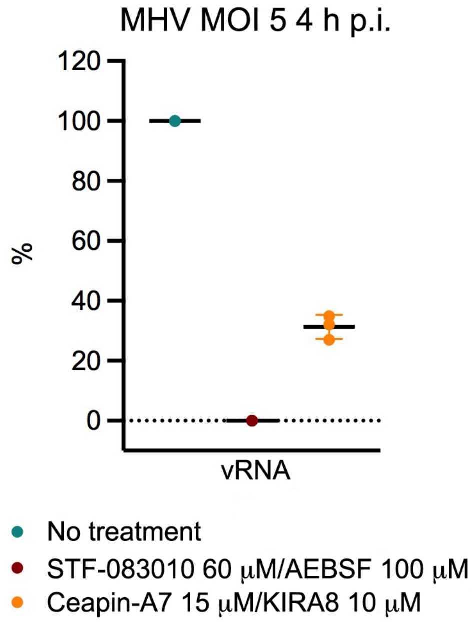

A potential inhibitory effect on early virus replication was tested by measuring viral RNA (vRNA) synthesis (primer binding site within the *N* gene, so will assay gRNA and all subgenomic RNAs) in 17 Cl-1 cells infected with MHV (MOI 5) at an early timepoint. As observed in the embedded figure, at 4 h p.i., RNA levels were reduced by 99% in cells treated with STF-083010 60 μM/AEBSF 100 μM (%N: 0.0639613) and by 69% in cells treated with the more specific UPR inhibitors, Ceapin-A7 15 μM/KIRA8 10 μM (%N: 31.31). This is highly consistent with the fact that we see reduced virus titres under these treatment conditions (Fig 3B and S11C).

CoV replication occurs in replication and transcription complexes (RTCs) which are detectable early in infection, from approximately 4 h p.i. (Gkogkas et al., 2008, PMDI: 18263603). The formation of RTCs is closely linked to large-scale manipulation of ER membranes to form convoluted membranes and an inter-connected network of double-membrane vesicles (DMVs), to which RTCs are anchored (Knoops et al., 2008, PMID: 18798692). The process of DMV formation is incompletely characterised, but it is entirely possible that inhibition of the UPR could interfere with DMV and/or RTC formation early in infection, with consequent inhibition of viral replication. For example, DMV formation during MHV infection is known to involve EDEM1 (Reggiori et al., 2010, PMID: 20542253), which is induced by XBP1-s as part of the UPR (Lee et al., 2003, PMID: 14559994). Inhibition of the UPR (particularly use of IRE1α inhibitors such as STF-083010 or KIRA8) could therefore disrupt formation of CoV replication complexes, and the observed reductions in vRNA abundance would be consistent with this.

Given that DMVs are derived from ER membranes, there may be other ways in which the UPR affects DMV formation and/or early viral replication. For example, VAPA, an ER protein involved in membrane trafficking, has been found in the RTC microenvironment and shown to be important for CoV replication (V'kovski et al., 2019, PMID: 30632963). VAPA has also been shown to interact with ER-localised ATF6 (Gkogkas et al., 2008, PMID: 18263603), providing one possible example of how changes in ATF6 localisation that result from UPR activation (or pharmacological inhibition) may therefore play a role in determining the composition of the RTC microenvironment and the efficiency of CoV replication.

In our study, the new experiments showing inhibited viral replication in the presence of the more specific inhibitor combination Ceapin-A7/KIRA8 (Fig 6, S11 Fig) further support the conclusion that the observed effects on viral replication are not indirect or off-target effects but demonstrate the complex inter-dependence of CoV replication and the UPR.

7. *Lines 435-439. Actually, no significant sequence homology is observed between SARS-CoV and SARS-CoV-2 8 protein. Therefore, differences in UPR activation might have multiple causes besides the presence of VLVVL motif.*

We agree – the VLVVL motif was intended just as one possible example, which has now been clarified in the text as well as adding the pairwise amino acid identity between the two proteins. Thank you for highlighting that this was not originally clear.

Reviewer #2:

1. *The authors use different time points for characterizing the UPR activation: 5 hpi (MHV) for RNA-seq and ribosome profiling, 2.5 – 8 hpi for later characterization. But then for SARS-CoV-2, UPR activation is characterized much later (24 & 48 hpi). Could the authors explain the relevance of the timing, especially in respect to the viral replication cycles for these viruses.*

As detailed in the response to Reviewer 1, in order to analyse UPR activation by CoVs, we were interested in finding a timepoint in which viral replication had reached the peak without cell viability being notably compromised. As previously published in Irigoyen et al., 2016 (PMID: 26919232), vRNA levels peak for MHV at 5 h p.i., whereas cytopathic effects (i.e. syncitium formation) appeared from 8 h p.i. onwards.

Regarding SARS-CoV-2 infection timings, we based these on published work (Chu et al., 2020, PMID: 32835326), showing that SARS-CoV-2 virus replication in Vero, Caco2 and Calu3 cells peaks between 24 h p.i. and 72 h p.i. In terms of cytopathic effect, Caco2 and Calu3 cells did not show substantial cell death in a time course of up to 120 h p.i., however, cell viability in Vero-infected cells decreased by 70% at 72 h p.i. These data were further confirmed by co-authors belonging to the Hale laboratory, where SARS-CoV-2 virus titres did not substantially increase after 24 or 48 h p.i. in Calu3 or Vero CCL81-infected cells.

Thus, the chosen times (24 and 48 h p.i.) were the most appropriate.

2. Induction of UPR markers on Western blot should be quantified more carefully and statistics should be provided. It is unclear if the Western blots are representative, and if so, how many other replicates were analyzed. Some of the reduction in signal seems small, for example the reduction in eIF2alpha phosphorylation in response to PERKi treatment in Fig. S5A. It would be important to provide a more quantitative measure for most of the Western blot data.

Unless indicated in the figure legend, all experiments were carried out in triplicate. Western blots shown are representative for the data analysed. In order to provide a more quantitative analysis of the UPR markers on western blots, bands corresponding to the different proteins have been quantified by densitometry and the fold-change relative to mock cells is indicated underneath the corresponding blot. Values represent the ratio of the band of interest relative to the loading control, normalised against the mock lane at that specific time point. This includes BiP, HERP, p-eIF2α and ATF6-Nt, with eIF2α and GAPDH as protein loading controls. PERK and ATF4 were not quantified because for PERK, phosphorylation state is the relevant feature as opposed to protein abundance, and for ATF4, the presence of multiple closely neighbouring bands makes accurate quantification difficult.

As indicated now by densitometry, in S5A Figure, p-eIF2α in non-treated infected cells is 3.75-fold greater than in mock-infected cells, and this value is reduced to 2.67 after PERKi treatment.

3. STF-083010 only leads to a very small reduction in XBP1 splicing (Fig. S5E, Fig. S6A-B) suggesting incomplete inhibition. No direct transcriptional targets of IRE1/XBP1s are analyzed as reporters of this pathway to confirm that the inhibitor can attenuate induction. For example, ERdj4 or P58ipk could be used (see lines 214). This would be important to confirm successful inhibition of IRE1 in the context of infection.

The splicing of *Xbp1* in S5E Fig was first assessed by densitometry, calculating the ratio between the spliced *Xbp1* (*Xbp1-s*) and the sum of *Xbp1-s* plus the unspliced *Xbp1* (*Xbp1-u*) (Shang et al., 2004, PMID 15063770). Quantified values of the *Xbp1-s* ratio under each condition, normalised by the ratio in mock-infected cells, have been added beneath the bands in the Figures.

In MHV-infected cells, the ratio between *Xbp1-s* and the sum of *Xbp1-s* and *Xbp1-u,* was 2.40-fold greater than the ratio in mock-infected cells. The mock-normalised *Xbp1-s* ratio was reduced to 1.82 upon IREi treatment, a reduction of ~25%. It is noteworthy, that this modest inhibition in *Xbp1* splicing by the IREi treatment is sufficient to significantly inhibit virus production (Fig 3A; ~2-fold inhibition of virus production).

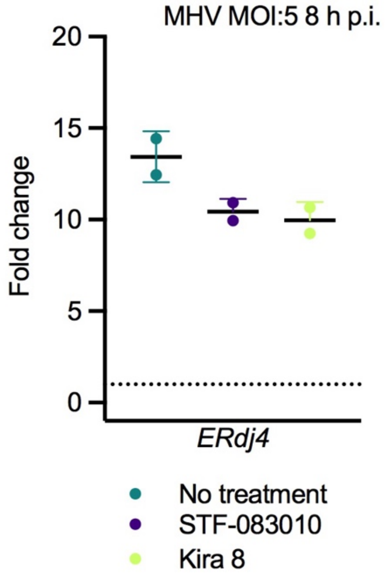

To further explore inhibition of the IRE1 branch of the UPR, we used an additional inhibitor, KIRA8. This reduced the *Xbp1-s* ratio (normalised by mock) from 7.30 in untreated MHV-infected cells to 1.30 in KIRA8-treated MHV-infected cells (S11B Fig), indicating near complete inhibition of the IRE1 pathway by KIRA8 during infection, and consequently, inhibition of virus production (S11C Fig). Further, qPCR of *ERdj4* in MHV-infected cells revealed that both inhibitors reduced the transcript abundance of this *Xbp1* target gene, corroborating *Xbp1* splicing inhibition (embedded Fig).

4. When constitutively overexpressing the recombinant S, Orf8, or Orf3a proteins, one would expect some of the UPR stress to be attenuated after some time. This should especially the case for the PERK branch and eIF2alpha phosphorylation. It is surprising and not clear from the Western blot (Fig. S7) that eIF2alpha remains phosphorylated at later time points. Overall, the characterization of UPR activation in response to Orf8 and S overexpression would be much clearer with bar graphs and more careful quantification of expression or phosphorylation changes.

As suggested by the reviewer, blots corresponding to S7 Fig have been quantified as previously indicated. This also includes the quantification of the ratio between the *Xbp1-s* and the sum of *Xbp1-s* plus *Xbp1-u* analysed by RT-PCR. These numbers correlate with the activation of the different UPR branches as indicated in lines 333-350 of the manuscript.

This confirms, as expected by the reviewer, that the UPR gets attenuated after some time. For example, phosphorylation of eIF2α in S prot-transfected cells at 36 h p.t. decreases compared to 24 h p.t., probably due to the activation of compensatory pathways to resolve the activation of the UPR. This correlates with the downregulation of *Bip* mRNA expression and the stabilisation of *Chop* mRNA by qPCR.

5. For SARS-CoV-2 infections, only the combinations of inhibitors were tested. In Fig. 5B, one striking observation is that it is mostly the combinations with AEBSF that are highly effective at reducing SARS-CoV-2 titers, raising a concern that much of the activity could be due to broad serine protease inhibition by AEBSF. What is the activity of individual inhibitors? This would be important to test. Why was such a low MOI (0.01) used for infections in Caco2 cells? Are the inhibitors still effective at higher MOI (as tested in Vero in Calu3)? Only cell metabolism (CellTiter Glo) is quantified in Calu3 and Caco2. Did the compounds similarly reduce cell proliferation as in other cells and could this contribute to the antiviral activity?

In response to the concern about the specificity of AEBSF:

As indicated by this reviewer, AEBSF is a highly promiscuous arylsulfonylchloride serine protease inhibitor that has a variety of pleiotropic effects besides ATF6 cleavage and it has the potential to be involved in the inhibition of several viral proteases (i.e. 3Cpro and PLpro). As seen in Fig 5C, the combination of the highly specific PERKi and IREi compounds was able to reduce virus production in Caco2 cells to the limit of detection, which is the same reduction that was seen for the combination of IREi with the more promiscuous AEBSF. This highlights that specific UPR inhibition can drastically reduce virus production for SARS-CoV-2 and suggests that promiscuity of AEBSF is not the major cause of viral replication inhibition.

In addition, to validate that the antiviral effect of AEBSF is specifically linked to inhibition of the ATF6 branch of the UPR, we tested a more selective pharmacological inhibitor of ATF6 cleavage, Ceapin-A7 (Gallagher et al., 2016, PMID: 27435960), as suggested by the reviewer. This compound was well tolerated by Vero CCL81, Calu3 and 17 Cl-1 cells (S10 Fig) and was verified to prevent ATF6 cleavage (Fig 6A) and transcriptional induction of *Grp94* (S11A Fig). Ceapin-A7 was able to significantly reduce replication of MHV and SARS-CoV-2 (Fig 6 and S11C and D). This shows that highly specific inhibition of the ATF6 UPR pathway inhibits viral replication and suggests this is the cause of the majority of the antiviral activity of AEBSF (discussed further in the next two paragraphs).

We have included a new section beginning at line 434 to explain this.

In response to the concern over reductions in cell proliferation potentially being responsible for the antiviral activity:

In addition to Ceapin-A7, we tested KIRA8, a specific kinase inhibitor that allosterically attenuates IRE1α RNase activity (Carlesso, et al., 2019, PMID: 31698846), in MHV- and SARS-CoV-2-infected cells. As well as its greater specificity (see above), the combination of Ceapin-A7 and KIRA8 was less cytotoxic than the IREi/AEBSF combination, and cell proliferation (and cell viability and metabolic activity) were above 80% of that of untreated cells in all cell types (S10 Fig). Ceapin-A7/KIRA8 produced similar (S11D Fig; SARS-CoV-2 in Calu3 cells) or even greater (Fig 6B; SARS-CoV-2 in Vero cells) reductions in virus titre than

IREi/AEBSF, indicating that the anti-viral activity of these UPR inhibitors is neither the result of cytotoxicity nor off-target/promiscuous activity.

As an additional note, we would like to indicate that for the revision experiments in which Ceapin-A7 and KIRA8 were tested against SARS-CoV-2 (MOI 5) in Calu3 cells (S11D Fig), a different stock of Calu3 cells, coming from a different laboratory, was used. As described above, these inhibitors (both individually and in combination) caused significant reduction in virus titres. Reductions were smaller than those observed using the previous stock of Calu3 cells at MOI 1 (Fig 5D). The higher MOI may contribute to the smaller titre reductions caused by these compounds in the new experiments, however, at this stage we cannot rule out genetic differences between these two clones of Calu3 cells that may affect virus replication. Nonetheless, in the new experiments, the extent of anti-viral activity was similar for the Ceapin-A7/KIRA-8 and STF-0830310/AEBSF combinations. This highlights the important point that the more specific (less toxic) drug combination inhibits virus production to the same extent as the more promiscuous combination, showing that the anti-viral effect does not result from off-target effects or cytotoxicity.

In response to the question of why we used a low MOI for the Caco2 experiments, and whether the inhibitors are still effective in Caco2 cells at high MOI:

The infections in Caco2 cells were performed in the Pirbright Institute (UK) relatively early during the pandemic and the amount of virus available at the time was only sufficient for infection of cells at low MOI (MOI 0.01). We thought it was important to expand upon these results to test other MOIs and other cell lines, however, due to the ongoing high demand for time/space in the BSL3 facility at the Pirbright Institute, availability for scheduling subsequent experiments there was limited. Therefore, further BSL3 experiments were performed in the Institute of Medical Virology (Switzerland), where we focused on using Calu3 and Vero cells at higher MOIs of 1-5.

In response to the question of what the activity of the individual inhibitors was against SARS-CoV-2:

Due to the strain on BSL3 facilities during the pandemic, we initially decided to select the most effective combination from the first panel of UPR inhibitors to test against SARS-CoV-2 and did not test the individual inhibitors. In response to these reviews, we tested the activity of the two newly added inhibitors, Ceapin-A7 and KIRA8, against SARS-CoV-2 both individually and in combination. Both inhibitors significantly inhibit SARS-CoV-2 replication in both Vero and Calu3 cells under single-drug treatment conditions, indicating that specific inhibition of either the ATF6 or the IRE1α pathway is sufficient to perturb virus production (Fig 6B, S11D).

6. It seems surprising that the inhibitors are so much more effective against SARS-CoV-2 than MHV given that UPR activation for both strains is comparable. In the absence of data highlighting that the antiviral activity of the inhibitors can be directly attributed to ATF6 and/or IRE1, another likely possibility is that SARS-CoV-2 is more sensitive to broad-spectrum protease inhibition by AEBSF.

Although MHV- and SARS-CoV-2-infected cells are able to activate the three different branches of the UPR, there are differences in virus replication cycle, especially in terms of duration, that could mean that the action, and therefore the inhibitory effect, of these compounds might have a stronger effect in a virus with a longer replication cycle such as SARS-CoV-2, in comparison to MHV.

Concerns about the specificity of AEBSF have been addressed in detail in the response to the previous comment. We cannot rule out that broad-spectrum protease inhibition by AEBSF

could have additional antiviral activity, although in our case the vast majority of the antiviral activity can be attributed to UPR inhibition. In fact, it would be very interesting to investigate the different steps at which AEBSF could act apart from S1P inhibition in order to determine extra potential antiviral targets.

Part III – Minor Issues: Editorial and Data Presentation Modifications

Reviewer #1:

1. *Regarding the activation of the UPR by coronavirus proteins, the paper by deDiego et al. (PLoS Pathog. 2011) showing that SARS-CoV E protein down-regulated the IRE-1 signaling pathway should be quoted (paragraph in lines 309-316).*

We have expanded the paragraph beginning at line 315 to include this.

2. *The discussion should mention that the phenotypes observed in cells overexpressing SARS-CoV-2 S proteins may not reflect their physiological functions in the real infection. To obtain physiologically relevant results, experiments with recombinant viruses with deletions or modification of the target viral proteins would be required.*

We have added this point in to the paragraph beginning at line 368.

Reviewer #2:

1. *The number of COVID-19 cases with severe diseases pathology (15%) seems high. This should be validated, and a reference provided.*

The number of cases with severe pathology varies considerably depending on the study – we have added clarification that the 15% figure likely represents the upper end of the range and provided references (Zhang et al., 2020, PMID: 32407459; and Wu and McGoogan, 2020, PMID: 32091533).

2. It seems puzzling that little attenuation of eIF2alpha phosphorylation is observed. Typically, during UPR activation, phosphorylation spikes at 2-6 hrs and decreases again relatively rapidly (for example Fig. 2A – Tm lanes). GADD34 is clearly induced as early as 5 hpi, which should lead to dephosphorylation. Could the authors explain this inconsistency?

Fig 2A has been quantified by densitometry. As indicated by the reviewer, eIF2α phosphorylation in tunicamycin-treated cells peaks at 5 h (10.1-fold greater than mock) and then decreases by 8 h (7.20-fold greater than mock) likely due to the action of GADD34. Our qPCR data revealed that the transcriptional induction of *Gadd34* upon tunicamycin treatment occurs at 5 h, suggesting that it takes time for *Gadd34* to accumulate, be translated and exert its effect on eIF2α phosphorylation.

Similarly, in MHV-infected cells, the peak of eIF2α phosphorylation occurs at 8 h p.i. (13.6-fold greater than mock) which correlates with the time it takes the virus to enter the cell and establish an infection. Then, phosphorylation of eIF2α decreases by 10 h p.i (10.01-fold greater than mock), in accordance with the transcriptional induction of *Gadd34* (Fig 2B).

3. In Fig. 2C, it looks like polysome amounts are low in both the mock and MHV treated conditions. Shouldn't the observed increase in 80S monosomes lead to a simultaneous decrease in polysomes?

Previous work with MHV (Hilton et al., 1986, PMID: 3009691) has revealed that virus infection increases the proportion of free 80S and reduces the levels of heavy polysomes, that is, there is a transition to lighter polysomes. This is precisely what we found, with an increase in both 80S and smaller polysomes. We appreciate that this is not immediately obvious from the image, but mouse 17-Cl1 cells produce very small quantities of polysomes in comparison to other cell lines. What is clear is that MHV infection generates a lot of free 80S ribosomes that are not mRNA associated, highly consistent with an initiation defect.

4. For the XBP1u/s RT-PCR reactions, why is there a third high-MW PCR product? Likewise, there seems to be high degree of background splicing activity in the absence of UPR stress? Do the authors have an explanation?

The third, high-MW PCR product that migrates above *Xbp1-u* is believed to represent a duplex of *Xbp1-s* and *Xbp1-u*, known in the literature as the "hybrid" band (*Xbp1-h*) (Shang et al., 2004, PMID 15063770).

cDNA derived from *Xbp1-s* and *Xbp1-u* transcripts is amplified by PCR with primers flanking the XBP1 spliced intron, and the products are distinguished from each other by a 26-bp difference in size when run on an agarose gel. However, this assay can also generate a third band, explained above, that migrates above double-stranded *Xbp1-u* on an agarose gel due to its bulkier structure. This phenomenon has been previously described (Chalmers et al., 2017, PMID: 29062910). To clarify this, we have included a comment in the legend of Fig 2D.

The higher background splicing in our assays seems to be more prevalent depending on the cell-type assayed (e.g. HEK-293T or Vero CCL81 cells) and is increased in experiments where cells have been in culture for longer periods.

5. The Western blot in Fig. 3C should be quantified. Why do some viral protein amounts increase with inhibitor treatments (for instance PERKi/ISRIB and ISRIB/IRE1i)?

As suggested by the reviewer, the western blot in Fig 3C has been quantified, indicating that in fact N protein translation in MHV-infected cells treated with the PERKi/ISRIB or the ISRIB/IREi combinations is increased 3.29- and 1.26-fold relative to untreated MHV-infected cells. We think that the inhibition of translation initiation that would normally be caused by eIF2α phosphorylation is alleviated by some inhibitor combinations which include ISRIB. This facilitates increased translation of viral proteins compared to the untreated conditions, but (as seen in Fig 3A) this does not correspond to an increase in the number of virus particles released. This disparity between abundance of viral proteins and production of infectious virus upon UPR inhibition could be for several reasons. CoVs assemble at intracellular membranes in the endoplasmic reticulum Golgi intermediate compartment (ERGIC), where they bud into the lumen and then are transported out of the cell by exocytosis. The ERGIC-53 protein, a cargo receptor required for glycoprotein trafficking within the early exocytic pathway, associates with the S protein in order to produce infectious virions (Klaus et al., 2013, PMID: 24237698). Moreover, ERGIC-53 expression is upregulated as a response to UPR activation (Spatuzza et al., 2004, PMID: 15292203). This provides one possible example of how inhibiting the UPR could have an effect on the availability of cargo proteins required for the

correct virion assembly and trafficking, even if more viral proteins are being synthesised as a result of less eIF2α phosphorylation.

We have addressed this concern in line 302 of the manuscript.

6. Upregulation of UPR protein markers often lags the transcriptional induction. That could explain the lack of BiP induction in Fig. 2F and S5A. Have the authors looked at later time points post infection? It would be good to blot for other ATF6-regulated genes, for example Grp94, PDIA4, calreticulin to corroborate RT-qPCR data showing ATF6 upregulation.

We agree it is a possibility that BiP would be detectable by western blot if a later timepoint was assayed, although the cytopathic effect after 10 h p.i. in MHV-infected cells is so extensive that cells cannot be harvested beyond this timepoint. We have now specified this in the manuscript in line 243.

However, as mentioned in the manuscript (line 241), BiP is known to be downregulated at the protein level during infection with some other members of the *Nidovirales* order. For example, it has been found that PRRSV (Order: *Nidovirales*, Family: *Arteriviridae*) encodes a protein that targets BiP for proteasomal degradation (Gao et al., 2019, PMID: 31738790). In addition, and it has been suggested (based on the observation that the BiP band on a western blot is weaker in HCoV-229E-infected cells than in mock-infected cells) that the human coronavirus HCoV-229E also downregulates BiP at the protein level (Shaban et al., 2020, bioRxiv doi 10.1101/2020.08.26.266304).

We do not have antibodies to the other ATF6 targets but, given that the induction of ATF6 targets is a transcriptional response, we think the results from both the qPCR (Fig 2E) and the RNASeq (Fig 1C in this manuscript and the embedded figure in this section) should provide a more accurate read-out for ATF6 activation than protein levels, which could be confounded by factors such as those mentioned above (or other, less specific effects such as different protein turnover rates).

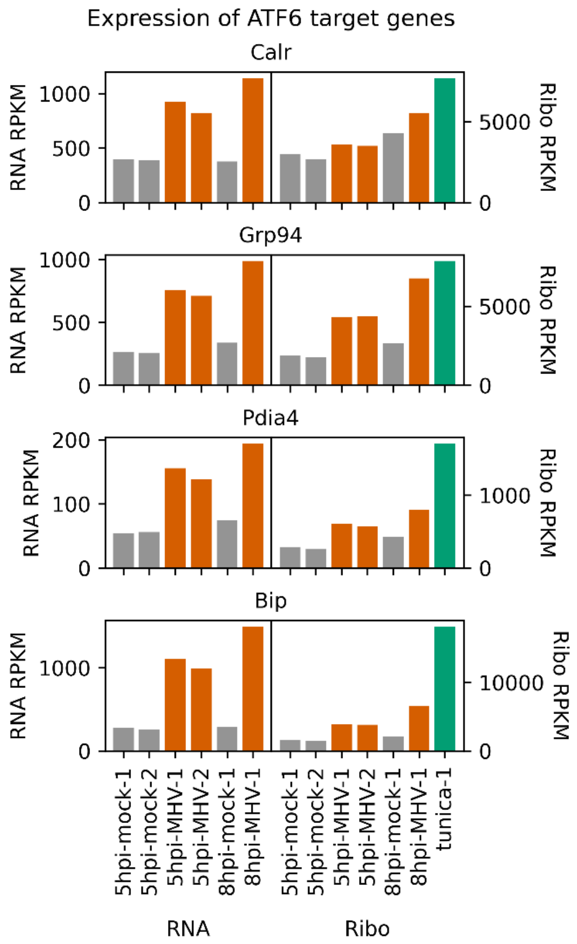

Expression of ATF6 target genes. RNASeq and RiboSeq read density was calculated in reads per kilobase of transcript per million mapped reads (RPKM), using reads mapping to NM_007591 (Calr), NM_011631 (Grp94), NM_009787 (Pdia4) or NM_022310 (Bip). Only positive sense reads were used and the normalisation for library size used total positive sense host-mRNA-mapping reads as the denominator.

7. Multiple cell models and MOI were used for SARS-CoV-2 infections, including Vero CCL81, Caco2 and Calu3 and MOI from 0.01 – 1. Was UPR activation by SARS-CoV-2 observed in Caco2 and Calu3 cells?

We have now characterised the UPR activation in Calu3 cells and included this data in a new panel (Fig 5B). However, for the experiments in Caco2 cells, the proportion of infected cells is expected to be 0.995%, calculated according to the Poisson distribution with an MOI 0.01. This is too low to observe substantial biochemical differences by Western blotting and RNA analysis (RT-PCR and qPCR).

8. It is not surprising that the authors found it challenging to detect ATF6 cleavage by Western blot in Fig. S2. The abcam ATF6 antibody clone is likely not good choice. Better ATF6 antibodies are: Anti-ATF6alpha antibody, mouse monoclonal (37-1) (for mouse) and Anti-ATF6 α antibody, mouse monoclonal (1-7) (for human) from BioAcademia.

Thank you, this will be useful information for future projects.
